# Cross‐cultural validation of the patient‐practitioner orientation scale among primary care professionals in Spain

**DOI:** 10.1111/hex.13135

**Published:** 2020-10-30

**Authors:** Lilisbeth Perestelo‐Pérez, Amado Rivero‐Santana, Ana Isabel González‐González, Carlos Jesús Bermejo‐Caja, Vanesa Ramos‐García, Débora Koatz, Alezandra Torres‐Castaño, Marta Ballester, Marcos Muñoz‐Balsa, Yolanda del Rey‐Granado, Francisco Javier Pérez‐Rivas, Yolanda Canellas‐Criado, Ana Belén Ramírez‐Puerta, Valeria Pacheco‐Huergo, Carola Orrego

**Affiliations:** ^1^ Evaluation Unit of the Canary Islands Health Service (SESCS) Tenerife Spain; ^2^ Health Services Research on Chronic Patients Network (REDISSEC) Tenerife Spain; ^3^ Centre for Biomedical Research of the Canary Islands (CIBICAN) Tenerife Spain; ^4^ Canary Islands Foundation and Institute for Health Research (FIISC) Tenerife Spain; ^5^ Primary Care Centre Vicente Muzas Community of Madrid Health Service Madrid Spain; ^6^ Institute of General Medicine Johann Wolfgang Goethe University Frankfurt‐am‐Main Germany; ^7^ Support Unit for Primary Care Community of Madrid Health Service Madrid Spain; ^8^ Nursing Department Autonomus University of Madrid Madrid Spain; ^9^ Avedis Donabedian Institute Autonomous University of Barcelona Barcelona Spain; ^10^ Nursing Department Complutense University of Madrid Madrid Spain; ^11^ Primary Care Centre Monóvar Community of Madrid Health Service Madrid Spain; ^12^ EAP Turó Barcelona Spain

**Keywords:** health‐care professionals, person‐centred care, PPOS, primary care, validation

## Abstract

**Background:**

In recent decades, many self‐report instruments have been developed to assess the extent to which patients want to be informed and involved in decisions about their health as part of the concept of person‐centred care (PCC). The main objective of this research was to translate, adapt and validate the Patient‐Practitioner Orientation Scale (PPOS) using a sample of primary care health‐care professionals in Spain.

**Methods:**

Baseline analysis of PPOS scores for 321 primary care professionals (general practitioners and nurses) from 63 centres and 3 Spanish regions participating in a randomized controlled trial. We analysed missing values, distributions and descriptive statistics, item‐to‐scale correlations and internal consistency. Performed were confirmatory factor analysis (CFA) of the 2‐factor model (*sharing* and *caring* dimensions), scale depuration and principal component analysis (PCA).

**Results:**

Low inter‐item correlations were observed, and the CFA 2‐factor model only obtained a good fit to the data after excluding 8 items. Internal consistency of the 10‐item PPOS was acceptable (0.77), but low for individual subscales (0.70 and 0.55). PCA results suggest a possible 3‐factor structure. Participants showed a patient‐oriented style (mean = 4.46, SD = 0.73), with higher scores for *caring* than *sharing*.

**Conclusion:**

Although the 2‐factor model obtained empirical support, measurement indicators of the PPOS (*caring* dimension) could be improved. Spanish primary care health‐care professionals overall show a patient‐oriented attitude, although less marked in issues such as patients’ need for and management of medical information.

## 1 INTRODUCTION

Person‐centred care (PCC) is currently advocated as the gold standard of health care, as opposed to a disease‐centred, paternalistic style.^1^ PCC implies an egalitarian relationship between health service users and professionals, in which patient values and preferences about their health care are placed at the core of the decision‐making process about diagnostic, therapeutic and lifestyle modification procedures. PCC aims to promote patient empowerment and involvement in the self‐management of their diseases, in a collaborative work of shared decision making with their health‐care providers, while respecting their autonomy and personal values. Establishing an emphatic and trust‐based relationship between patients and professionals and facilitating high‐quality communications are thus necessary requisites for PCC to take hold.^2^ PCC relies not only on ethical arguments about people's rights to autonomy and personal independence in a democratic society, but also on its potential benefits for health and the sustainability of health systems. It is expected that more active, informed and empowered patients will be able to achieve better self‐management of their conditions and will improve adherence to therapeutic plans discussed and agreed with their health‐care providers. As well as improving coordination and continuity of services, this could result in improved health outcomes and greater resource use efficiency.^3^, ^4^, ^5^

In the last decades, many self‐report instruments have been developed to assess the extent to which patients want to be informed and involved in decisions about their care, the most widely used of which are the Control Preference Scale^6^ and the Autonomy Preference Index.^7^ Research has shown that most patients desire a collaborative or autonomous role in their medical decisions and that many of them do not feel as involved as they would want.^3^, ^8^ Furthermore, less perceived involvement or a mismatch between preferred and experienced involvement has been shown to be related to poorer satisfaction, adherence and quality of life.^9^, ^10^, ^11^, ^12^, ^13^ Although not all variations in patients’ perceptions of being involved are caused by an actual deficit in health‐care professionals’ behaviour, the above results clearly reinforce the need to enhance PCC and improve patient participation. To achieve this aim, health‐care providers must develop certain communication and social skills—and an obvious prerequisite is to hold favourable beliefs and attitudes towards this model of care. Consequently, assessing these attitudes becomes a relevant issue in the research and implementation of PCC in routine practice and in academic curricula. While a number of instruments have been developed for that purpose, this is lesser extent than for patients.^14^, ^15^, ^16^

One such instrument is the Patient‐Practitioner Orientation Scale (PPOS), developed in the United States by Krupat et al.^17^ It consists of 18 items grouped in 2 subscales of 9 items each: *sharing* assesses attitudes about whether health‐care professionals should share information, decisions and power with their patients, while *caring* assesses the degree to which professionals should show empathy and warmth and treat patients as whole persons. Higher scores in the PPOS have been shown to be associated with more patient‐centred behaviours in consultations,^18^ while congruence between patients and physicians’ orientations has been demonstrated to be associated with greater patient satisfaction,^19^, ^20^, ^21^ trust and endorsement of physicians,^19^ and fewer referrals.^22^ The PPOS, which has been translated from English to several other languages,^23^, ^24^, ^25^ has been widely used to assess preferences for patient orientation among health‐care professionals, medical students and even patients, since it is applicable to the general population.^26^, ^27^, ^28^, ^29^, ^30^ However, few studies have reported the psychometric properties of the instrument or, more specifically, its factorial validation.^31^, ^32^, ^33^

Since (to our knowledge) no studies have been published on use of the PPOS in Spain, our aim was to translate, adapt and carry out a psychometric validation of this scale using a sample of health‐care professionals.

## 2 METHODS

This study analyses PPOS baseline data for participants, who were recruited during 2016, for a cluster randomized controlled trial that aimed to assess the impact of a virtual community practice intervention on health‐care professionals (general practitioners and nurses) at the primary care level.^34^ Primary care centres from 3 Spanish autonomous communities (Canary Islands, Catalonia and Madrid) were contacted via their managers and invited to participate. Centres were randomly selected, while a balanced north/south geographical representation was maintained within each region. In‐person meetings were held in each centre to explain the study in detail to interested professionals. Those who agreed to participate signed the informed consent and received a password to access a web interface where they could fill out the PPOS questionnaire. Participants’ allocation to the intervention group or control group was only disclosed after the questionnaire was completed.

### 2.1 Measures

#### 2.1.1 Patient‐practitioner orientation scale

This 18‐item scale measures the orientations of patients and health‐care professionals regarding the patient‐practitioner relationship. The scale is scored on a 1‐6 Likert scale (totally disagree‐totally agree). Items, except for 9, 13 and 17, are written in a physician‐oriented style; scoring is therefore reversed in such a way that a higher score indicates a patient‐oriented style. Scores for the overall scale (18 items) and *sharing* and *caring* subscales (9 items each) are divided by their corresponding number of items and thus range between 1 and 6.

### 2.2 Sociodemographic and professional data

Data were collected on age, sex, profession (general practitioner or nurse), years’ experience, tutorship of medical residents/medical or nursing students (yes/no), and patients attended per day.

### 2.3 Questionnaire translation

As the methodological model for Spanish translation of the PPOS, we used the guidelines on cross‐cultural adaptation of self‐reported measure developed by Beaton et al,^35^ based on five steps as follows:

1. A pair of bilingual translators, competent in both English and Spanish, independently translated the original questionnaire from English to Spanish.
2. Working with the original questionnaire and both translation versions, the translators synthesized the translation after reaching a consensus on the translation of words, phrases and items.
3. Five primary care physicians and nurses independently tested the cultural appropriateness, representativeness and content validity of the translated instrument, rating the degree that each item reflected the concept that it was intended to measure. The same professionals also rated the understandability of the translated instrument and the semantic and content equivalence of the Spanish version with the English original.
4. To ensure that meaning accurately reflected the English original, the Spanish version was back‐translated by a different pair of bilingual translators working blind to the original English version.
5. In a final equivalence testing step, the back‐translation was compared with the original instrument by the study directors in Spain (LPP, AIGG, CJBG and CO) and, after some minor revisions, the Spanish version was considered ready to use.

The final Spanish version of the PPOS was pre‐tested on first twelve adult patients attended at the two primary care centre participants in this study and their responses were analysed to identify whether any modifications were necessary, which resulted not to be the case.

### 2.4 Statistical analyses

The distribution and descriptive statistics for the items (missing values, frequencies, means, standard deviations, asymmetry and kurtosis) were calculated. Floor and ceiling effects for each item were defined as more than 85% of participants scoring 1 (totally disagree) and 6 (totally agree), respectively. Also calculated were the mean inter‐item correlations, corrected item‐to‐scale correlations and Cronbach's α after excluding each item. In order to assess whether the data fit the 2‐factor model proposed for the scale (*sharing* and *sharing*), a confirmatory factor analysis (CFA) was performed. Missing values were handled by using full information maximum likelihood estimation, which does not require the imputation of missing values, but uses all the available data to estimate population parameters.^36^ However, in the presence of non‐normal data this technique can produce negatively biased standard errors, leading to an erroneous rejection of the null hypothesis. For this reason, we first assessed non‐normality by means of Yuan, Lambert and Fouladi's normalized estimate of multivariate kurtosis, applicable to data with missing values^37^ (a value outside the range −3,3 is indicative of multivariate kurtosis). In the case of non‐normality, standard errors were corrected using the robust method proposed by Yuan and Bentler.^38^

The model was refined by repeating the analysis after excluding items with non‐significant coefficients or low R^2^ values. Its fit was then assessed by means of the chi‐square test (or the Yuan‐Bentler's scaled chi‐square,^38^ in the case of performing robust estimation); since this statistic is very sensitive to sample size, we calculated several other recommended goodness‐of‐fit indices^39^, ^40^, ^41^: χ^2^/*df* ratio, root mean square error of approximation (RMSEA) with a 90% confidence interval (CI), Tucker‐Lewis index (TLI), comparative fit index (CFI) and Bollen's incremental fit index (IFI). We considered the following thresholds for acceptable and good values, respectively^39^, ^40^, ^41^: under 3 and 2 for the χ^2^/*df* ratio; under 0.08 and 0.05 for the RMSEA; and above 0.90 and 0.95 for the TLI, CFI and IFI. If the model did not obtain an acceptable fit even after being refined, we carried out a principal component analysis (PCA) to explore other potential factorial models.

Finally, we assessed associations of the obtained scales with participants’ sociodemographic and professional characteristics. Due to the clustered nature of the study design (ie professionals clustered into centres), we used a 2‐level mixed multiple regression model, with fixed effects for professionals (level 1) and random effects for centre (level 2), to adjust for correlated observations within the clusters. Analyses were performed with SPSS 21.0 and EQS 6.2 software.

## 3 RESULTS

Contacted were 113 primary care centres, 9 and 41 of which declined participation and failed to respond, respectively, leaving 63 centres to be included (25 in the Canary Islands, 18 in Catalonia and 20 in Madrid). These contributed 321 health‐care professionals (mean 5.1, range 1‐28). Table 1 shows sociodemographic and professional characteristics of the professionals. Mean age was 47.7 years (SD 8.8), and 76% were female. Over half (59%) were general practitioners, and the remaining 41% were nurses. Mean years’ experience was 22.0 (SD 8.84), mostly in primary care (mean 17.7; SD 8.92). The professionals attended a mean of 29 patients per day (SD 11.3), and 25.5% had tutored residents/medical and nursing students.

**Table 1:** Sociodemographic and professional characteristics of participants

	N = 321
Age (mean, SD)	47.7 (8.79)
Age	
26‐35	24 (7.5%)
36‐45	114 (35.5%)
46‐55	105 (32.7%)
56‐66	78 (24.3%)
Female	243 (75.7%)
Physicians/nurses	190 (59.2%)/131 (40.8%)
Years’ experience	22.0 (8.84)
Years in primary care	17.7 (8.92)
Tutor	82 (25.5%)
Patients/day	29.4 (11.3)

### 3.1 Item analyses

For the 18 items, 25 participants (7.8%) missed between 1 and 3 items (20, 2 and 3 participants missed 1, 2 and 3 items, respectively). Item #8 had 15 missing values (4.7%), whereas 9 more items had between 1 and 4 missing values (Table 2). There were no ceiling or floor effects for any item. Distributions were asymmetric, with most participants stating some level of disagreement (slightly, moderately or totally disagreed) with the physician‐oriented style. Item #9, written in patient‐oriented terms (*Patients should be treated as if they were partners with the doctor, equal in power and status*), disagreement was high (80%). Favourable or less critical responses with the physician‐oriented style were obtained for 4 items, specifically, item #5 (*Patients should rely on their doctors’ knowledge and not try to find out their conditions on their own*), item #8 (*Many patients continue asking questions even though they are not learning anything new*), item #10 (*Patients generally want reassurance rather than information about their health*) and item #18 (*When patients look up medical information on their own, this usually confuses more than it helps*).

**Table 2:** Missing values, distribution of responses and descriptive statistics of PPOS items

	Missing (%)	A/D^a^ (%)	Mean^b^ (SD)	Asym.	Kurt.
1. The doctor is the one who should decide what gets talked about during a visit.	0	30/70	4.37 (1.4)	0.498	−0.915
2. Although health care is less personal these days, this is a small price to pay for medical advances.	0.3	24/76	4.63 (1.4)	0.780	−0.529
3. The most important part of the standard medical visit is the physical exam.	0	31/69	4,12 (1.3)	0.413	−0.655
4. It is often best for patients if they do not have a full explanation of their medical condition.	0	11/89	5.11 (1.2)	1.441	1.653
5. Patients should rely on their doctors’ knowledge and not try to find out their conditions on their own.	0	36/64	3.93 (1.5)	0.366	−0.957
6. When doctors ask a lot of questions about a patient's background, they are prying too much into personal matters.	0	3/ 97	5.59 (0.8)	2.876	10.14
7. If doctors are truly good at diagnosis and treatment, the way they relate to patients is not that important.	0	4/96	5.54 (0.9)	2.578	7.665
8. Many patients continue asking questions even though they are not learning anything new.	4.7	52/48	3.51 (1.4)	−0.035	−0.840
9. Patients should be treated as if they were partners with the doctor, equal in power and status.	0.6	20/80	2.32 (1.4)	0.966	−0.060
10. Patients generally want reassurance rather than information about their health.	0.9	44/56	3.67 (1.3)	0.051	−0.965
11. If a doctor's primary tools are being open and warm, the doctor will not have a lot of success.	0.6	13/87	4.89 (1.2)	1.089	0.644
12. When patients disagree with their doctor, this is a sign that the doctor does not have the patient's respect and trust.	0.9	27/73	4.29 (1.4)	0.589	−0.610
13. A treatment plan cannot succeed if it is in conflict with a patient's lifestyle or values.	0.3	72/28	4.40 (1.6)	−0.746	−0.655
14. Most patients want to get in and out of the doctor's office as quickly as possible.	0	21/79	4.51 (1.4)	0.909	−0.042
15. The patient must always be aware that the doctor is in charge.	0.3	12/88	5.10 (1.2)	1.420	1.454
16. It is not that important to know a patient's culture and background in order to treat the person's illness.	0	4/96	5.54 (0.9)	2.939	9.710
17. Humor is a major ingredient in the doctor's treatment of the patient.	1.2	86/14	4.66 (1.2)	−0.893	0.683
18. When patients look up medical information on their own, this usually confuses more than it helps.	0.3	67/33	3.02 (1.3)	−0.343	−0.478

Note

Asym., asymmetry; Kurt., kurtosis.

^a^ Agree/disagree, collapsing the 3 categories for each (slightly, moderately, strongly).

^b^ Higher scores indicate more patient‐oriented style (reversed scores for all items except #9, #13, #17).

### 3.2 Dimensionality and internal consistency

Mean inter‐item correlation was 0.15 (median 0.14). The 3 items written in a patient‐oriented style (#9, #13 and #17) obtained the lowest mean correlations (0.08, 0.07 and 0.09, respectively). Table 3 shows the item‐to‐scale correlations and Cronbach's alphas after excluding each item. The 3 mentioned items showed the lowest correlations (under 0.14). The remaining value ranges were 0.26‐0.51 (*sharing*), 0.17‐0.38 (*caring*) and 0.18‐0.48 (overall). Excluding items #9, #13 and #17 increased alphas from 0.72 to 0.77 (overall), from 0.66 to 0.72 (*sharing*) and from 0.48 to 0.56 (*caring*); these items were therefore excluded from subsequent analyses.

**Table 3:** Item‐to‐scale correlations and PPOS overall and subscale internal consistency

Items	Sharing (α = 0.660)		Caring (α = 0.477)		Total (α = 0.722)	
	Item to scale	α without item	Item to scale	α without item	Item to scale	α without item
#1	0.41	0.615			0.45	0.694
#4	0.39	0.623			0.45	0.698
#5	0.51	0.587			0.48	0.689
#8	0.44	0.608			0.41	0.699
#9	−0.08	0.722			−0.03	0.743
#10	0.44	0.611			0.46	0.695
#12	0.37	0.625			0.37	0.703
#15	0.26	0.648			0.29	0.711
#18	0.35	0.631			0.31	0.710
#2			0.27	0.418	0.37	0.703
#3			0.26	0.425	0.36	0.704
#6			0.33	0.425	0.35	0.710
#7			0.38	0.405	0.41	0.705
#11			0.24	0.434	0.25	0.715
#13			0.10	0.499	0.13	0.731
#14			0.19	0.457	0.18	0.723
#16			0.17	0.458	0.24	0.715
#17			0.04	0.501	0.11	0.726

We carried out a CFA for the 2‐(correlated) factor model. We used the maximum likelihood (with missing values) estimation method, with robust standard errors due to the non‐normal distribution of the data (multivariate kurtosis normalized estimate 25.5). All items obtained significant coefficients and, as shown in Table 4, the χ^2^/*df* ratio (2.3) and RMSEA values (0.063, 90% CI: 0.052‐0.075) were acceptable, while the TLI, CFI and IFI values were unsatisfactory. We repeated the analysis excluding items #14, #15 and #16, with R^2^ values under 0.15; however, the improvement did not achieve the acceptability thresholds. Excluding 2 more items (#11 and #18, thus leaving 6 items for *sharing* and 4 for *caring*) yielded acceptable values for the χ^2^/*df* ratio (2.0), RMSEA (0.056, 90% CI: 0.035‐0.075), CFI and IFI (0.92 for both), while bringing TLI to the limit of that threshold (0.89). When errors for items #6 and #7 were allowed to covariate (based on their significant standardized residual covariance), all indices except the chi‐square indicated a good fit, while the TLI was acceptable (0.93). A one‐factor model retaining those 10 items yielded minimal differences with the 2‐factor solution (Table 4).

**Table 4:** Fit statistics from PPOS confirmatory factor analysis

N = 321	Χ^2^; *df* (p)^a^	Χ^2^/*df*	RMSEA (90% CI)	TLI	CFI	IFI
2‐factor (15 items)	202.6; 87 (<0.001)	2.3	0.063 (0.052 − 0.075)	0.777	0.815	0.821
2‐factor (12 items)	128.6; 51 (<0.001)	2.5	0.068 (0.053 − 0.083)	0.815	0.857	0.861
2‐factor (10 items)	65.5; 32 (<0.001)	2.0	0.056 (0.035 − 0.075)	0.888	0.920	0.923
2‐factor modified^b^ (10 items)	50.3; 31 (0.016)	1.6	0.043 (0.017 − 0.064)	0.934	0.955	0.956
One‐factor (10 items)	70.3; 34 (<0.001)	2.1	0.056 (0.037 − 0.075)	0.886	0.914	0.916
One‐factor modified^b^ (10 items)	53.4; 33 (0.014)	1.6	0.042 (0.018 − 0.063)	0.935	0.952	0.954

Note

CFI, comparative fix index; *df*, degrees of freedom; IFI, incremental fit index; RMSEA, root mean square error of approximation; TLI, Tucker‐Lewis fit index.

^a^ Yuan‐Bentler's scaled chi‐squared statistic.

^b^ Allowing the covariance of errors of items 6 and 7.

The CFA only showed a good fit of the data after exclusion of 8 items: 3 *sharing* items (#9, *Patients should be treated as if they were partners with the doctor, equal in power and status*, #15, *The patient must always be aware that the doctor is in charge* and #18, *When patients look up medical information on their own, this usually confuses more than it helps*) and 5 *caring* items (#11, *If a doctor's primary tools are being open and warm, the doctor will not have a lot of success*, #13, *A treatment plan cannot succeed if it is in conflict with a patient's lifestyle or values*, #14, *Most patients want to get in and out of the doctor's office as quickly as possible*, #16, *It is not that important to know a patient's culture and background in order to treat the person's illness* and #17, *Humour is a major ingredient in the doctor's treatment of the patient*). The resulting scales had Cronbach's alphas of 0.70 (*sharing*), 0.55 (*caring*) and 0.77 (overall) and means (SDs) of 4.12 (0.88), 4.97 (0.74) and 4.46 (0.73), respectively.

We performed several PCA with varimax rotation to explore other potential latent structures, including only the 15 items phrased in a physician‐oriented style. The analysis yielded 4 components with eigenvalues greater than one. When a fifth factor was extracted, only a single item showed high loading (#14). In the 4‐factor solution, the last factor included only 2 items with high loadings (#16, #15), not clearly related semantically speaking. The 3‐factor solution is shown in Table 5. The first, second and third components include 5 items on information, 6 items on the patient‐physician relationship and 4 items favouring technical aspects of medicine and an asymmetric relationship between patient and professional, respectively. Items #4, #11 and #14 saturated above 0.30 in 2 or 3 components. When 2 components were extracted, the above‐mentioned first and third components collapsed into a single dimension (not shown in the table).

**Table 5:** Principal component analysis of the PPOS items (excluding items #9, #13 and #17)

	C1	C2	C3	h^2^
#18	**0.77**	−0.10	−0.06	0.37
#5	**0.68**	0.15	0.21	0.50
#8	**0.58**	−0.00	0.30	0.45
#10	**0.53**	0.18	0.23	0.44
#12	**0.47**	0.19	0.23	0.52
#16	0.09	**0.72**	−0.15	0.53
#6	0.05	**0.70**	0.20	0.52
#7	−0.01	**0.55**	**0.47**	0.43
#15	0.12	**0.53**	0.15	0.36
#14	**0.34**	**0.41**	−**0.32**	0.31
#11	−0.02	**0.39**	**0.38**	0.32
#2	0.24	0.07	**0.66**	0.55
#3	0.21	−0.05	**0.64**	0.61
#1	0.28	0.22	**0.49**	0.39
#4	**0.33**	**0.33**	**0.47**	0.30
Explained variance	15.5%	14.5%	13.9%	
Cronbach's α	0.68	0.60^*^	0.63	

Note

Kaiser‐Meyer‐Olkin 0.82; Bartlett's sphericity test: χ^2^ = 799.2, *P* < .001.

Loadings above 0.30 in bold.

^*^ Items #16, #6, #7, #15.

### 3.3 Associations with sociodemographic and professional variables

Multilevel mixed regression models did not point to any significant association between the 10‐item PPOS overall or its subscales and age, sex, profession, years’ experience, tutorship, or patients attended per day.

## 4 DISCUSSION

For our sample of Spanish health‐care professionals in the primary care sector, the proposed structure of the PPOS with 2 correlated factors (representing the *sharing* and *caring* dimensions) only obtained an acceptable fit to the data after excluding 8 items (almost half of the total scale with 18 items). Inter‐item correlations were observed to be low overall; the 3 items phrased in a patient‐oriented style were the most uncorrelated, pointing out to a possible methodological effect that has also been observed in other studies for 2 of these items.^31^, ^32^ The PCA also pointed to poor functioning of several of the remaining items, which loaded similarly on more than a single component. Of the extracted components, 2 (C1 and C2) can be partially assimilated, respectively, to *sharing* and *caring*. The main difference between the CFA and PCA results was the functioning of items #2 (*Although health care is less personal these days, this is a small price to pay for medical advances*) and #3 (*The most important part of the standard medical visit is the physical exam*). These items were retained in the *caring* 4‐item subscale in the CFA; in the PCA, they formed the third component in the 3‐factor solution and the first component in the 2‐factor solution, thus seeming to some extent to be independent of the relational aspects of the patient‐physician interaction represented by C2. From this perspective, the items may be mere indicators of the importance attributed to technical aspects of medicine and, therefore, may not necessarily be incompatible with the interpersonal or socio‐affective aspects of health care as represented by *caring*.

Few studies in recent years have reported on the psychometric properties of the PPOS since its initial development and psychometric testing, mainly focused on criterion validity.^19^, ^20^, ^26^ Those studies have also pointed to certain psychometric limitations of the instrument. Wang et al^31^ in reporting results with a mixed sample of physicians (20%) and patients in China, documented a very poor fit to the data in a CFA with the 18 items; depuration of the scales retained 11 items, 6 for *sharing* and 5 for *caring*, although the excluded items were not the same as in our study. In a German study, the scale was also reduced, in that case to 12 items.^42^ In a study conducted in Mali, Hurley et al (2018)^43^ also obtained a very poor model fit for the 18 items for a sample of medical students, while depuration of the scale by means of exploratory factor analysis did not identify an interpretable structure that fit well to the data. Pereira et al^33^ in a validation study in Brazil, observed low internal consistency values for the overall score (0.61) and for the *sharing* (0.49) and *caring* (0.46) scores for a sample of medical students/residents (33%) and patients; a CFA with the 18 items yielded unsatisfactory values for the CFI (0.84) and TLI (0.81), although the χ^2^/*df* ratio, RMSEA and SRMR values were acceptable. Another study with medical students/health‐care professionals (29%) and patients in Sri Lanka, although it did not analyse the factor structure of the scale, found low internal consistency values (0.50‐0.63) and item‐to‐scale correlation values.^32^ Although the comparisons with our study are not straightforward due to differences in samples, the psychometric limitations identified in those studies from different countries around the world are similar to those found by us, suggesting that our results are not sample‐specific but due in fact to non‐optimal functioning of the questionnaire.

Regarding the scores obtained, most participants showed a patient‐oriented style, with mean values for the 10‐item version similar to or higher than observed for other published samples of health‐care professionals. The *caring* dimension obtained a higher score, indicating a greater preference for the socio‐affective side of PCC than for sharing information and power. This result, while corroborated by several studies,^29^, ^31^, ^32^, ^44^, ^45^ could depend on the cultural background of professionals or their medical specialty.^46^, ^47^ In our sample, s*haring* subscale items that obtained more physician‐oriented responses were related to the patients’ need for information and searches for themselves. These results may indicate professionals’ concerns about patients’ exposure to unreliable information, a risk that is not negligible nowadays with the great amount of information available online, but also reflects a possible underestimation of patients’ real needs for accurate information about their conditions (and not just reassurance).

We found no significant associations for the overall or subscale scores with sociodemographic or professional variables. Studies that have used the PPOS to identify predictors of patient orientation in health‐care professionals mostly observed no significant associations with age or sex,^26^, ^31^, ^48^, ^49^ although a Greek study did find that younger physicians were more patient‐oriented in the *sharing* dimension.^50^ As for differences between physicians and nurses, like us, Zhumadilova et al^30^ observed no significant differences in Kazakhstan, whereas Chan et al^51^ found higher scores for physicians in Malaysia. Given the wide geographic distribution of countries in which the PPOS has been used, future studies should compare scores and correlates of the PPOS for different countries and geographic regions, since differences in cultural backgrounds and health systems may be influencing the attitudes of health‐care professionals to PCC and its correlates.

Assessing these attitudes is an important issue for research, clinical practice and educational purposes, since PCC cannot be successfully implemented if health‐care professionals are not well disposed to the principles of this model of care. Consequently, the availability of measurement instruments with good psychometric properties is a basic requisite to be able to appropriately assess the level of patient‐centredness in our health systems, identify areas for improvement and evaluate the effect of interventions to promote PCC and shared decision making. This study adds new evidence on the psychometric characteristics of the PPOS, an instrument widely used worldwide.

## 5 LIMITATIONS

A limitation of this study is that, although the sample was not small, it was not large enough to be randomly split into 2 subsamples: to explore the latent structure and to act as a validation sample (recommended rules of thumb such as a ratio of items/participant of at least 1/10 or a minimum of 200 participants would not have been met). Another possible limitation is that we did not impute missed data. However, the rate of missing values was low and we used appropriate techniques to deal with them. Yet another limitation is that we did not apply empirical methods to determine the number of components to extract in the PCA; nonetheless, the inspection of the different alternatives showed that solutions based on 2 or 3 components were the most plausible, given the low number of items included and the low factor loadings in the fourth and fifth extracted components.

## 6 CONCLUSION

The similarity of our results with those of recent validation studies supports the conclusion that the PPOS has psychometric limitations. Although the 2‐factor dimensions of *sharing* and *caring* obtained support from the data, measurement indicators could be improved, especially those of the *caring* factor.

## CONFLICT OF INTEREST

The authors state that there is no conflict of interests.

## AUTHOR CONTRIBUTIONS

LPP conceived the study, participated in its design, is the PI of the Canary Islands region and collaborated in writing this manuscript. AIGG conceived the study, participated in its design, is the PI for the Madrid region and wrote up the final manuscript. CO conceived the study, participated in its design, coordinates the trial, is the principal investigator (PI) for the Catalonia region and collaborated in writing this manuscript. CBCJ conceived the study, participated in its design, is co‐PI for the Madrid region and collaborated in writing this manuscript. LPP and ARS analysed data and wrote drew up the initial manuscript draft. VRG, DK, ATC, MB, MMB, YRG, YCC and ABRP contributed to writing the manuscript. All authors made substantial contributions to the revising of the manuscript and approved the final version.

## Data Availability

All data generated or analysed during this study are included in this published article.
